# Taraxerone inhibits M1 polarization and alleviates sepsis-induced acute lung injury by activating SIRT1

**DOI:** 10.1186/s13020-024-01002-z

**Published:** 2024-11-14

**Authors:** Lang Deng, Weixi Xie, Miao Lin, Dayan Xiong, Lei Huang, Xiaohua Zhang, Rui Qian, Xiaoting Huang, Siyuan Tang, Wei Liu

**Affiliations:** 1 Xiangya Nursing School, Central South University, Changsha, 410013 Hunan China; 2 Occupational Disease Department, Hunan Prevention and Treatment Institute for Occupational Diseases, Changsha, 410021 Hunan China

**Keywords:** Acute lung injury, SIRT1, Taraxerone, Oxidative stress, Inflammation, Macrophage polarization

## Abstract

**Background:**

Acute lung injury (ALI) is the most lethal disease associated with sepsis, and there is a lack of effective drug treatment. As the major cells of sepsis-induced ALI, macrophages polarize toward the proinflammatory M1 phenotype and secrete multiple inflammatory cytokines to accelerate the disease process through nuclear factor kappa-B (NF-κB) and NLR family pyrin domain containing 3 (NLRP3) inflammasome signaling pathways. Taraxerone, the main component of the Chinese medicinal *Sedum*, possesses numerous biological activities. However, uncertainty remains regarding the potential of taraxerone to protect against sepsis-induced ALI. This study aimed to investigate the effects and mechanisms of taraxerone against ALI.

**Methods:**

An animal model for ALI was established by cecal ligation and puncture and treated with taraxerone via intraperitoneal administration. The protective effect of taraxerone on the lungs was analyzed using H&E staining, dihydroethidium staining, ELISA kits, cell counting, myeloperoxidase kit, malondialdehyde kit, glutathione kit, superoxide dismutase kit and flow cytometry. Western blotting, RT-PCR, flow cytometry, co-immunoprecipitation, and immunofluorescence were used to investigate the regulatory of taraxerone on SIRT1.

**Results:**

Our study demonstrates for the first time that taraxerone can activate SIRT1 in macrophages, promoting SIRT1 activity. This activation inhibited the NF-κB signaling pathway primarily through the dephosphorylation and deacetylation of p65. Simultaneously, taraxerone disrupted the NLRP3 inflammasome signaling pathway, thereby alleviating M1 polarization of macrophages and mitigating sepsis-induced pulmonary inflammation and oxidative stress. In vivo, EX527 was used to validate the anti-inflammatory and anti-oxidative stress effects of taraxerone mediated by SIRT1.

**Conclusion:**

SIRT1-mediated anti-inflammatory and anti-oxidative stress effects may be important targets for taraxerone in treating ALI.

**Supplementary Information:**

The online version contains supplementary material available at 10.1186/s13020-024-01002-z.

## Introduction

The World Health Organization declared sepsis a particular concern in 2017 [1]. Its substantial mortality risk alongside its capacity to impact various organs was highlighted [2]. Notably, the lungs appear particularly vulnerable, potentially leading to acute lung injury (ALI) or acute respiratory distress syndrome (ARDS) [3]. ARDS is the most common severe manifestation of multi-organ dysfunction syndrome and a key factor in sepsis morbidity and mortality [4]. The exact pathogenesis of ALI is unclear. As a commonly used animal model of ALI, cecal ligation and puncture (CLP) rapidly causes sepsis, which in turn triggers an endotoxin-triggered inflammatory cascade in vivo, resulting in an inflammatory storm, that induces lung injury [5, 6]. Mechanical ventilation is still the treatment of choice in clinical due to the lack of effective therapeutic drugs. However, its side effects are nonnegligible. The death rate from ARDS remains high, with a severe impact on survival of patients [7]. Accordingly, it is urgently necessary to discover effective drugs for ALI that will improve patients’ outcomes.

The most significant cells controlling the local inflammatory environment and inflammatory response in the lungs are the alveolar macrophages (AMs), which play a significant role in sepsis-induced ALI by controlling inflammation and oxidative stress [8]. Under the stimulation of lipopolysaccharide (LPS), macrophages polarize toward the proinflammatory phenotype M1 and subsequently release numerous and multiple inflammation cytokines (interleukin (IL)-1β, IL-6, tumor necrosis factor-alpha(TNF-α), and IL-12), recruiting neutrophils, monocytes, and other cells, thereby causing an amplified cascade of inflammatory responses [9–11]. M1 macrophages also lead to NO overproduction by increasing the expression of inducible nitric oxide synthase(iNOS) genes, resulting in tissue damage [12]. Activation signals of signal transducer and activator of transcription 1(STAT1) or Toll-like receptor 4(TLR4) lead to activation of nuclear factor kappa-B (NF-κB), which triggers transcription of M1-related genes and promotes the polarization of M1 phenotype macrophages [13, 14]. Reactive oxygen species (ROS) enhance the activation of these pathways [15–17]. Studies have revealed that ROS concentration-dependently promote M1 macrophage polarization [18, 19].

Sirtuin1 (SIRT1) is a deacetylase-active, NAD ( +)-dependent metabolic regulatory enzyme directly associated with the metabolic status of the body [20]. It has been reported that SIRT1 activation exerts a deacetylating effect on downstream molecules and inhibits protein phosphorylation simultaneously [21]. SIRT1 inhibits the activation of NF-κB and its downstream responses, and inhibition of SIRT1 activity is followed by increased expression of inflammatory cytokines [22, 23]. SIRT1 also reduces endoplasmic reticulum stress, mitochondrial dysfunction, and excessive ROS production in sepsis-induced ALI models by inhibiting oxidative stress [24]. Therefore, SIRT1 may exert anti-inflammatory and anti-oxidative stress effects that play inhibitory roles in the development of ALI.

Taraxerone (Tar), an effective ingredient of the Chinese folk herb *Sedum*, is isolated from herbal extract [25]. Studies have shown that taraxerone has significant biological effects, such as immunomodulatory, antiallergic, anti-inflammatory, antibacterial, hypoglycemic, and antitumor [26–28]. It has been shown that taraxerone effectively reduces the increase in IL-6 content in LPS-induced RAW264.7 and may inhibit macrophage activation [26]. Moreover, it has been shown that taraxerone has strong anti-oxidative stress activity and dose-dependently reduces ROS production in neutrophils [29]. Taraxerone has anti-inflammatory and anti-oxidative stress effects, but its specific mechanism remians unclear. In our study, we found originally the protective function of taraxerone against sepsis-induced ALI in mice and further investigated the effect of taraxerone via SIRT1 to determine the mechanism of its anti-inflammatory and anti-oxidative stress effects.

## Methods

### Experimental animals

8 weeks male C57BL/6 mice were purchased from the Animal Centre of Central South University, Changsha, China. Before the experiment, all the mice were adaptively fed for one week and fasted for 24 h before the procedure.

After being anesthetized with pentobarbital sodium, the mice received cecal ligation and puncture [6]. The sham group underwent the same surgical procedure except for the ligation and puncture. The animal experiment was divided into 2 programs, the first program was aimed to verify the effects of taraxerone on ALI and the second program was aimed to verify the mechanism of taraxerone. The mice in the first program were injected saline or taraxerone (1, 6, 30 mg/kg) 2 h before the CLP operation, and the mice in the second program were injected saline or EX527 (10 mg/kg) 2 h before the taraxerone (0 mg/kg or 30 mg/kg) administered, after that the CLP model was constructed. The mice in the first program were randomly divided into 5 groups and treated as follows. (1) CON group, saline (*i.p.*) + sham operation; (2) CLP group, saline (*i.p.*) + CLP; (3) CLP + 1 mg/kg group, Tar (1 mg/kg, *i.p.*) + CLP; (4) CLP + 6 mg/kg group, Tar (6 mg/kg, *i.p.*) + CLP; (5) CLP + 30 mg/kg group, Tar (30 mg/kg, *i.p.*) + CLP. The mice in the second program were randomly divided into 4 groups and treated as follows. (1) CON group, saline (*i.p.*) + saline (*i.p.*) + sham operation; (2) CLP group, saline (*i.p.*) on + saline (*i.p.*) + CLP; (3) CLP + Tar group, saline (*i.p.*) + Tar (30 mg/kg, *i.p.*) + CLP; (4) CLP + Tar + EX527 group, EX527 (10 mg/kg, *i.p.*) + Tar (30 mg/kg, *i.p.*) + CLP. Every group had 16 mice. All the solutions were intraperitoneally (*i.p.*) administered.

To compare the effects of taraxerone and the positive control drug dexamethaso [30] on sepsis-induced acute lung injury in mice, the mice were randomly divided into four groups and treated as follows: (1) CON group, saline (*i.p.*) + saline (*i.p.*) + sham operation; (2) CLP group, saline (*i.p.*) + saline (*i.p.*) + CLP; (3) CLP + Tar group, saline (*i.p.*) + taraxerone (30 mg/kg, *i.p.*) + CLP; (4) CLP + DEX group, dexamethasone (5 mg/kg, *i.p.*) + CLP. Each group consisted of 16 mice. All solutions were administered via intraperitoneal injection (*i.p.*).

To verify the effect of taraxerone on CLP, the mice were reandomly divided into three groups and treat as follows: (1) CON group, saline (*i.p.*) + saline (*i.p.*) + sham operation; (2) CLP group, saline (*i.p.*) + saline (*i.p.*) + CLP; (3) CLP + Tar group, saline (*i.p.*) + taraxerone (30 mg/kg, *i.p.*) + CLP. Each group consisted of 16 mice. All solutions were administered via intraperitoneal injection (*i.p.*).

Taraxerone (National Institutes for Food and Drug Control, China, purity ≥ 98%) was diluted in the vehicle consisting of saline containing 5%(v/v) dimethyl sulfoxide (DMSO)(Macklin, China). All experiments adhered to the International Guiding Principles for Biomedical Research Involving Animals and received approval from the Ethics Committee of the Center for Scientific Research with Animal Models at Central South University (Certificate No.CSU-2022–0586; Changsha, China).

### Collection and analysis of Bronchoalveolar lavage fluid (BALF)

Mice were sacrificed 12 h after the CLP operation and BALF was collected with cooled PBS. The fluid was centrifuged at 1500r/min at 4 °C for 10 min. The BALF was divided into several parts: One part was used for flow cytometry to detect M1 macrophages proportion (Elabscience, China) and ROS level (Thermo Fisher, USA), and the other part was used for cell classification counting with Wright-Giemsa staining. In addition, BALF samples can be used to detect total protein content (Beyotime, China) and cytokine levels after treatment.

### Histological analysis

The lung tissue was divided into several parts, one of which was fixed with 4% (w/v) paraformaldehyde to prepare paraffin-embedded tissue sections, and stained with hematoxylin&eosin (H&E) according to the procedure to observe the pathological changes.

### Lung wet/dry (W/D) ratios

Lung tissue was harvested 12 h after CLP operation, promptly weighed to determine the ‘wet’ weight, and subsequently dried in a 56 °C oven for 48 h until reaching a consistent weight to obtain ‘dry’ weight. The ratio of wet weight to dry weight was employed to obtain the edema in lung tissue.

### ELISA assay

The treated BALF and supernatant was used to determine the secretion levels of IL-6, TNF-α and IL-1β by enzyme-linked immunosorbent assay (ELISA) kit (Elabscience, China). The treated serum was used to determine the secretion levels of TNF-α, IL-1β and IL-6 by ELISA kit (Jianglai, China). The absorbance value of each hole was measured and analyzed at a wavelength of 450 nm.

### Extraction and culture of primary peritoneal macrophages

Mice were intraperitoneally injected with 3 mL 3% thioglycolate (Sigma-Aldrich, USA) 4 days in advance, and were lavaged with 15 mL pre-cooled RPMI 1640 (Gibco, USA) to collect the cells. The cell suspension was centrifuged at 4 °C and 1500 rpm for 10 min, then lysed erythrocytes and centrifuged and re-suspended. The cell suspension was inoculated into the cell culture plate according to the density of 1 × 10^6^ cells per pore, and the cells adhered to the wall 2 h later for follow-up treatment.

Primary peritoneal macrophages were cultured in an incubator at 37 °C and 5%CO_2_ with a complete medium containing 10% fetal bovine serum (Gibco, USA) and 1% penicillin/streptomycin (Procell, China). We treated cells with LPS (1 μg/mL, Sigma-Aldrich, USA) and taraxerone (10 μM, 30 μM, 50 μM) for 12 h to investigate the effect of taraxerone on the inflammatory environment of macrophages. The protein content in the nucleus was detected after LPS and taraxerone treatment for 3 h. In addition, cells were pretreated with EX527 (5 μM/mL) for 2 h, and then treated with LPS (1 μg/mL) and taraxerone (50 μM) for 12 h to investigate the effects of SIRT1.

### Cell viability assay

Cell viability was measured using the Cell Counting Kit 8 (Abcam, USA) as directed by the manufacturer. Primary macrophages were inoculated into 96-well plates and cultured with a complete medium containing 10%FBS to a growth density of 70%. Taraxerone of different concentrations (0 μM, 1 μM, 3 μM, 10 μM, 30 μM, 50 μM) was changed for 24 h. After that, RPMI 1640 solution containing CCK8 was incubated at 37 °C. We measured the absorbance value at 450 nm and analyzed it.

### Measurement of MPO, GSH, SOD, and MDA levels

We grind the collected lung tissue into a lung homogenate, dissolve it in the extraction buffer, and tested with Myeloperoxidase (MPO) assay kit、Malondialdehyde (MDA) assay kit、Superoxide Dismutase (SOD) assay kit、Reduced glutathione (GSH) assay kit (Jiancheng Bioengineering Institute, China) according to the manufacturer’s instructions.

Primary macrophages were prepared into cell suspension or cell precipitates and were dissolved in the extraction buffer, and MDA content, SOD activity, and GSH were detected and analyzed with the above kits.

### Dihydroethidium (DHE) staining

The lung tissue was prepared into frozen slices and return to normal temperature during testing. First, tissues were treated with cleaning solution for 5 min and then incubated with dyeing solution for 30 min against the light. Then, slices were washed twice with PBS. After staining with DAPI in the dark, slices were washed with PBS for 30 min. Finally,they were examined under fluorescence microscope.

### ROS production assay

Primary macrophages precipitated or cell precipitates from BALF were re-suspended in H2DCFDA (Thermo Fisher, USA) and incubated at 37 °C for 30 min in the dark. After washing with PBS 3 times, the production of reactive oxygen species was determined under fluorescence microscope. The excitation wavelength is 488 nm and the emission wavelength is 525 nm. The fluorescence intensity was quantified using ImageJ software. In addition, after the cells were incubated according to the above steps, flow cytometer (BD LSRFortessa, USA) was used to detect ROS production and FlowJo software was used for data analyses.

### M1 macrophages determination

The percentage of M1 macrophages in BALF was measured through flow cytometry. Cells were stained with PE-conjugated anti-mouse F4/80 (Elabscience, China) and FITC-conjugated CD80 [31–33]. Flow cytometer (BD LSRFortessa, USA) was used to detect M1 macrophage proportion and FlowJo software was used for subsequent analysis.

### Immunofluorescence

During immunofluorescence staining, primary macrophages underwent triple washing with PBS and fixation with 4% paraformaldehyde for 15 min. After penetration with 0.5%Triton X-100, the cells were exposed to goat serum blocking solution (ZSGB Bio, China). Following this, overnight incubation with P65 antibody (Abcam, UK) at 4 °C. After washing with PBST 3 times, the cells were subjected to a 1 h fluorescent secondary antibody (Proteintech, China), and stained with DAPI (Biosharp, China). The nuclear translocation of P65 was observed under a fluorescence microscope (Zeiss Apotome).

### Co-Immunoprecipitation

For the detection of the interaction between SIRT1 and P65, cells underwent a triple wash with PBS and were subsequently lysed with lysis buffer containing PMSF (Solarbio, China) on ice for 1 h. Cells were harvested and centrifuged at 12,000 rpm for 10 min at 4 °C. The supernatant was incubated with Dynabeads™ Protein G (Invitrogen, 10004D) for 3 h at 4 °C and then centrifuged. Subsequently, Dynabeads were separated with DynaMag™-2 (Invitrogen, 12321D). SIRT1 polyclonal antibody (Proteintech, China) was added to the supernatant overnight at 4 °C. Dynabeads were separated with DynaMag™-2 by washing them 3 times with lysis buffer. The SIRT1-binding proteins were pulled down and analyzed by western blotting.

### Molecular docking

Protein and compound data was acquired from RCSB PDB (https://www.rcsb.org/) and PubChem (https://pubchem.ncbi.nlm.nih.gov/). Deleted water molecules, added hydrogen atoms, and completed docking at AutoDock Vina, as well as Phyton and MGLTools [34, 35]. PyMol software and Protein–Ligand Interaction Profiler were performed for the calculation of the distance between taraxerone and residues of SIRT1, and data visualization [36].

### Total RNA preparation and real-time PCR

RNA from lung tissues and primary macrophages was extracted by Trizol (Thermo Fisher Scientific, USA), and the purity of RNA was determined by Varioskan LUX multifunctional enzyme marker (Thermo Fisher Scientific, USA) to ensure that A260/280 ratio was in the 1.8–2.2 range. It was reverse transcribed into cDNA using a reverse transcription kit (Thermo Fisher Scientific, USA) according to the manufacturer's instructions. cDNA was extracted by SYBR Green (Promega, USA) and Bio-Rad CFX96 Touch Real-Time PCR Detection System (Bio-Rad, USA) for real-time qPCR assay. The primer was synthesized by Shanghai Sangong Biotechnology Co., LTD., and the sequences are as follows shown in Table 1.

**Table 1:** Sequences of the primers used to quantitate gene expression

Gene	Forward primer(5′-3′)	Reverse primer(5′-3′)
TNF-α	AGCCCCCAGTCTGTATCCTT	CTCCCTTTGCAGAACTCAGG
IL-6	CTGGGGATGTCTGTAGCTCA	CTGTGAAGTCTCCTCTCCGG
IL-1β	GGGCCTCAAAGGAAAGAATC	TACCAGTTGGGGAACTCTGC
IL-18	TCGGGAAGAGGAAAGGAACC	TTCTACTGGTTCAGCAGCCA
IL-12p40	CAGAAGCTAACCATCTCCTGGTTTG	TCCGGAGTAATTTGGTGCTTCACA
iNOS	CATTGATCTCCGTGACAGCC	CATGCTACTGGAGGTGGGTG
NLRP3	TACGGCCGTCTACGTCTTCT	CGCAGATCACACTCCTCAAA
Pro Caspase-1	TCATTTCCGCGGTTGAATCC	CCAACAGGGCGTGAATACAG
ASC	GACAGTACCAGGCAGTTCGT	AGTCCTTGCAGGTCAGGTTC
β-actin	TTCCAGCCTTCCTTCTTG	GGAGCCAGAGCA GTAATC

### Western blotting analysis

Proteins were extracted from lung tissue or primary macrophages and protein concentrations were measured using Pierce™ BCA Protein Assay Kit (Thermo Fisher Scientific, USA). The proteins were separated by 10% SDS-PAGE gel electrophoresis and transferred to a PVDF membrane (Bio-Rad, USA). After being blocked with skim milk for 2 h, the cells were incubated overnight with antibodies of the target protein at 4 °C. The proteins were incubated at room temperature for 2 h with secondary antibody (1:5000, Proteintech, China). Bands were visualized by using Luminate™Crescendo chemiluminescent horseradish peroxidase substrate (Millipore, USA). Scanned the band by using GeneGnome XRQ imager (Syngene, UK). The gray value was measured and analyzed using ImageJ software. The primary antibodies used in this study are shown in Table 2.

**Table 2:** Primary antibody sources and dilutions

Antibodies	Source	Dilution ratio
Anti-NF-κB P65 monoclonal antibody	Proteintech	1:1000
Anti-Pho- NF-κB P65 monoclonal antibody	CST	1:1000
Anti-IκBα monoclonal antibody	Abcam	1:2500
Anti-Pho-IκBα polyclonal antibody	Abcam	1:10,000
Anti-Ace- NF-κB P65 polyclonal antibody	Affinity	1:500
Anti-NLRP3 monoclonal antibody	CST	1:2000
Anti-Pro Caspase-1/p10/p20 monoclonal antibody	Abcam	1:1000
Anti-ASC/TMS1 monoclonal antibody	ABclonal	1:500
Anti-SIRT1 polyclonal antibody	Proteintech	1:1000
Anti-β-actin polyclonal antibody	Proteintech	1:1000
Anti-Histone H3 monoclonal antibody	Abcam	1:1000

### Determination of respiratory function

After being anesthetized, mice were subjected to tracheotomy and tracheal intubation. We used the BUXCO Mouse Respiratory Function Test System (Max II, USA) to measure the breathing frequency and dynamic compliance.

### Caspase-1 activity assay

Primary macrophages were centrifuged and collected after LPS and taraxerone treatment. After being resuspended with Reagent 2, the activity of caspase-1 was detected for absorbance of 450 nm according to the caspase-1 activity assay kit(Soloarbio, China).

### Statistical analysis

All the above data were represented by mean ± SD and analyzed by GraphPad Prism 9.0 software. Comparisons between three or more groups were performed using One-way analysis of variance and Student-Newman-Kill (SNK) tests. P < 0.05 was considered statistically significant.

## Results

### Anti-inflammatory effects of taraxerone on mice with sepsis-induced ALI

**Fig. 1:**
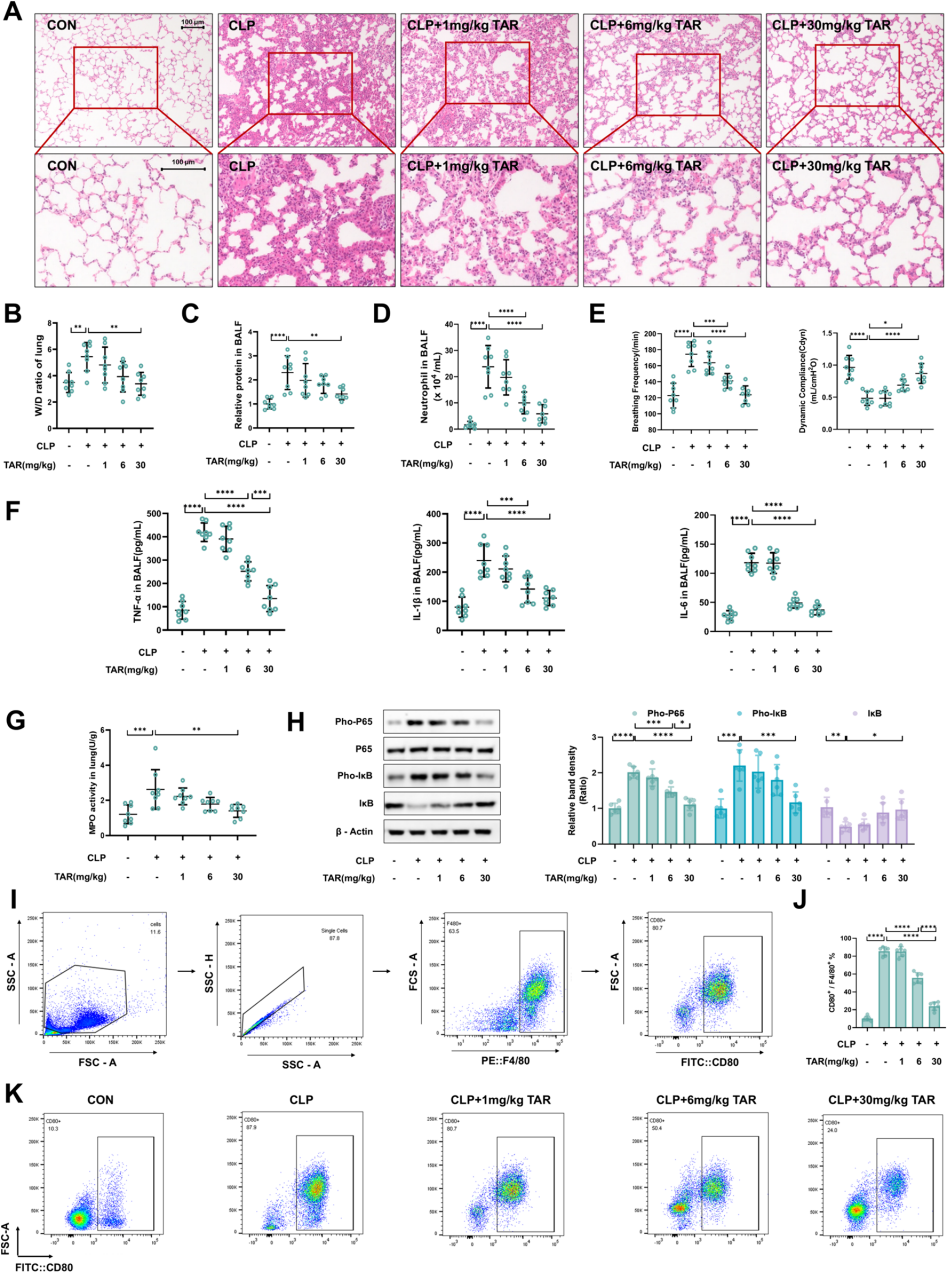
Taraxerone ameliorates sepsis-induced acute lung injury in mice. Taraxerone (0, 1, 6, and 30 mg/kg) was administered intraperitoneally, and the lung tissues and BALFs were collected 12 h after the CLP model was established. **A** H&E staining was used to detect histopathological changes in lung tissue. The bar represented 100 μm. **B** The W/D ratio of lung tissue was measured to determine pneumonedema. **C** BALFs were collected for total protein content and **D** count of neutrophils. **E** The data of breathing frequency and dynamic compliance were detected and collected by BUXCO system. **F** TNF-α, IL-1β, and IL-6 in BALFs were detected by ELISA. **G** MPO activity in lung tissue. **H** The Pho-P65, P65, Pho-IκB, and IκB levels in lung tissues were determined using western blotting. **I** Schematic diagram of the process of screening M1 macrophages by flow cytometry. **J**–**K** The change in M1 macrophage proportion was detected using flow cytometry. Data are presented as mean ± SD (n ≥ 6 in each group). *P < 0.05, **P < 0.01, ***P < 0.001, ****P < 0.0001

First, to confirm the protective effect of taraxerone against ALI, we observed histopathological changes of lungs. The lung tissue of mice undergoing CLP showed obvious pathological changes, such as the accumulation of inflammatory cells and thickening of alveolar walls. However, these changes were significantly inhibited by treatment of 6 and 30 mg/kg taraxerone (Fig. 1A). Similarly, pretreatment with 30 mg/kg taraxerone significantly lowered the wet-to-dry (W/D) ratio and levels of protein in bronchoalveolar lavage fluid (BALF), indicating that taraxerone reduces lung edema and leakage of proteins (Fig. 1B, C). In addition, by cell counting in BALFs, we observed that neutrophil count after treatment with 6 or 30 mg/kg taraxerone were below the CLP-treated alone group (Fig. 1D). Furthermore, we determined the protective effects of taraxerone by testing the variation of pulmonary function. Taraxerone could decrease the breathing frequency, and improve the dynamic compliance of mice with sepsis (Fig. 1E). Moreover, the anti-inflammatory effect of taraxerone is that it also reduces the production of cytokines such as IL-6, IL-1β, and TNF-α in lung tissue and BALFs, and inhibits the increase of myelloperoxidase (MPO) activity (Fig. 1F, G). The mRNA levels of IL-6, IL-1β, and TNF-α were also detected (Fig. S1). In addition to this, taraxerone inhibits the NF-κB pathway, a classical inflammation pathway, by the up-regulation of IκB and down-regulation of Pho-IκB and Pho-P65 (Fig. 1H). Since macrophage polarization is an essential part of the progression of ALI and M1 macrophages are the major effector cells of the inflammation, we examined the ratio of M1 macrophages in BALFs using flow cytometry. The results showed that the proportion of M1 macrophages in BALFs from mice in the CLP-treated alone group was apparently exceed the control group, whereas the proportion of M1 macrophages in the 6 and 30 mg/kg taraxerone pretreatment groups was effectively decreased (Fig. 1I, K). In addition, we compared the anti-inflammatory effects of dexamethasone as a positive control with taraxerone. Results from H&E staining, W/D ratio, BCA analysis, cell counts, MPO levels, lung function, ELISA of IL-1β, IL-6, and TNF-α indicated that both taraxerone and dexamethasone exhibit effective therapeutic effects on sepsis-induced ALI, with no significant differences observed between the two treatments (Fig. S2). Besides, We also tested the effect of taraxerone on CLP through the H&E staining of spleen, live, and kidney, and IL-1β, IL-6, and TNF-α levels of serum. The results showed that taraxerone could ameliorate the inflammatory injury in mice (Fig. S3). Taken together, taraxerone alleviated ALI induced by sepsis, and there were significant differences between the treatment with 6 and 30 mg/kg taraxerone.

### Anti-oxidative stress effects of taraxerone on mice with sepsis-induced ALI

**Fig. 2:**
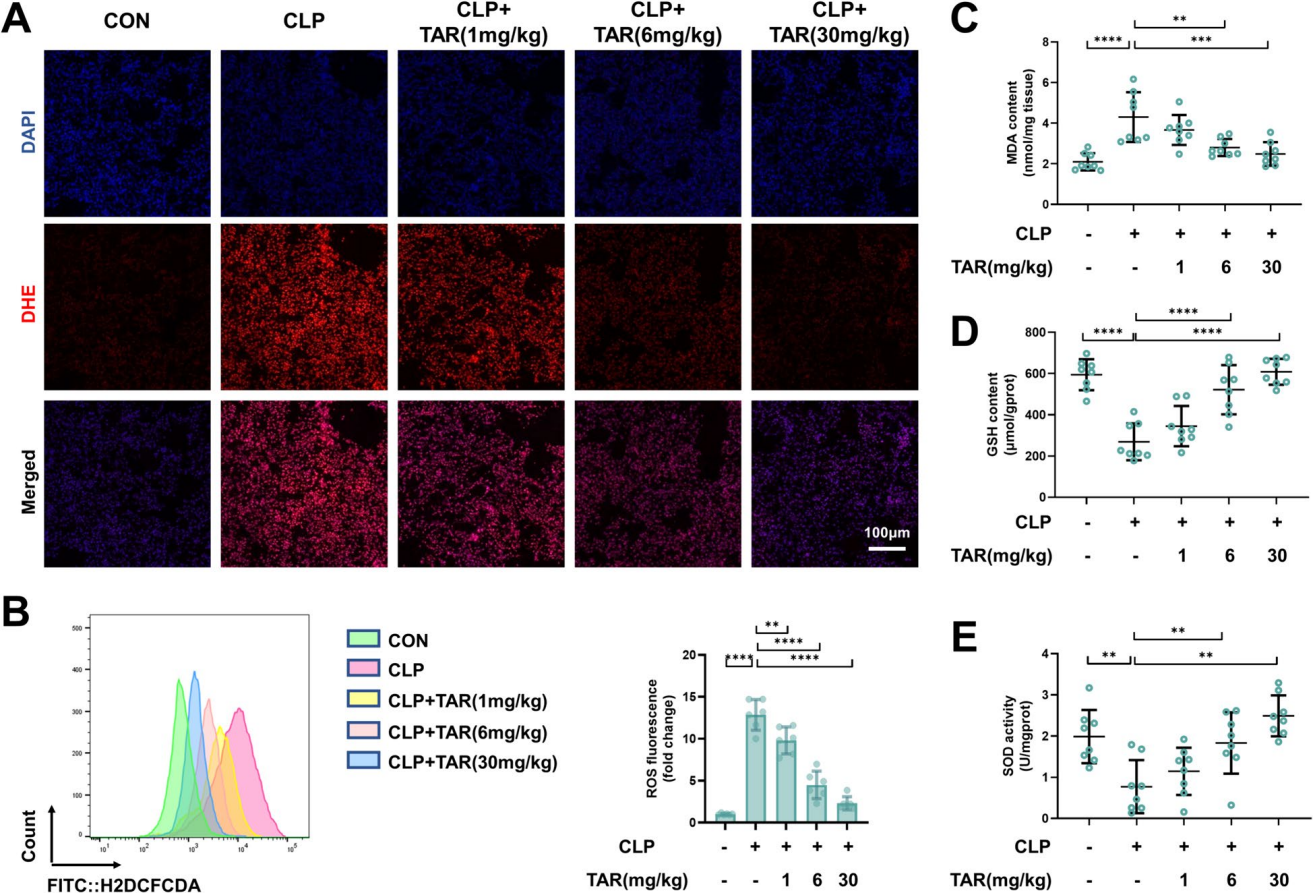
Taraxerone mitigates oxidative stress in mice with sepsis-induced ALI. Mice were pretreated with taraxerone (1, 6, and 30 mg/kg) or saline for 2 h before cecal ligation and puncture, and BALFs and pulmonary tissues were gathered and quantified 12 h later. **A** DHE staining was used to detect ROS levels in tissues. The bar represented 100 μm. **B** ROS production in BALFs was detected using flow cytometry and analyzed using FlowJo. **C**–**E** MDA content, GSH content, and SOD activity in lung were detected. Data are presented as mean ± SD (n ≥ 6 in each group). **P < 0.01, ***P < 0.001, ****P < 0.0001

Oxidative damage is an important contributor to sepsis-induced ALI. ROS levels in the lung tissues were detected using dihydroethidium (DHE) staining. The results revealed that ROS levels were considerably higher in the CLP-treated group. In contrast, the excessive ROS increase was inhibited by taraxerone treatment (Fig. 2A). Similarly, flow cytometry was utilized to determine ROS levels in mice BALF, and the results were in line with those obtained using DHE staining (Fig. 2B). Furthermore, taraxerone pretreatment markedly inhibited the sepsis-induced increase in malondialdehyde (MDA) content (Fig. 2C). In addition, taraxerone increased the glutathione (GSH) levels and superoxide dismutase (SOD) activity (Fig. 2D, E). According to our results, taraxerone preconditioning mitigated oxidative stress in mice with sepsis-induced ALI.

### Taraxerone blocks LPS-induced inflammatory responses in primary macrophages

**Fig. 3:**
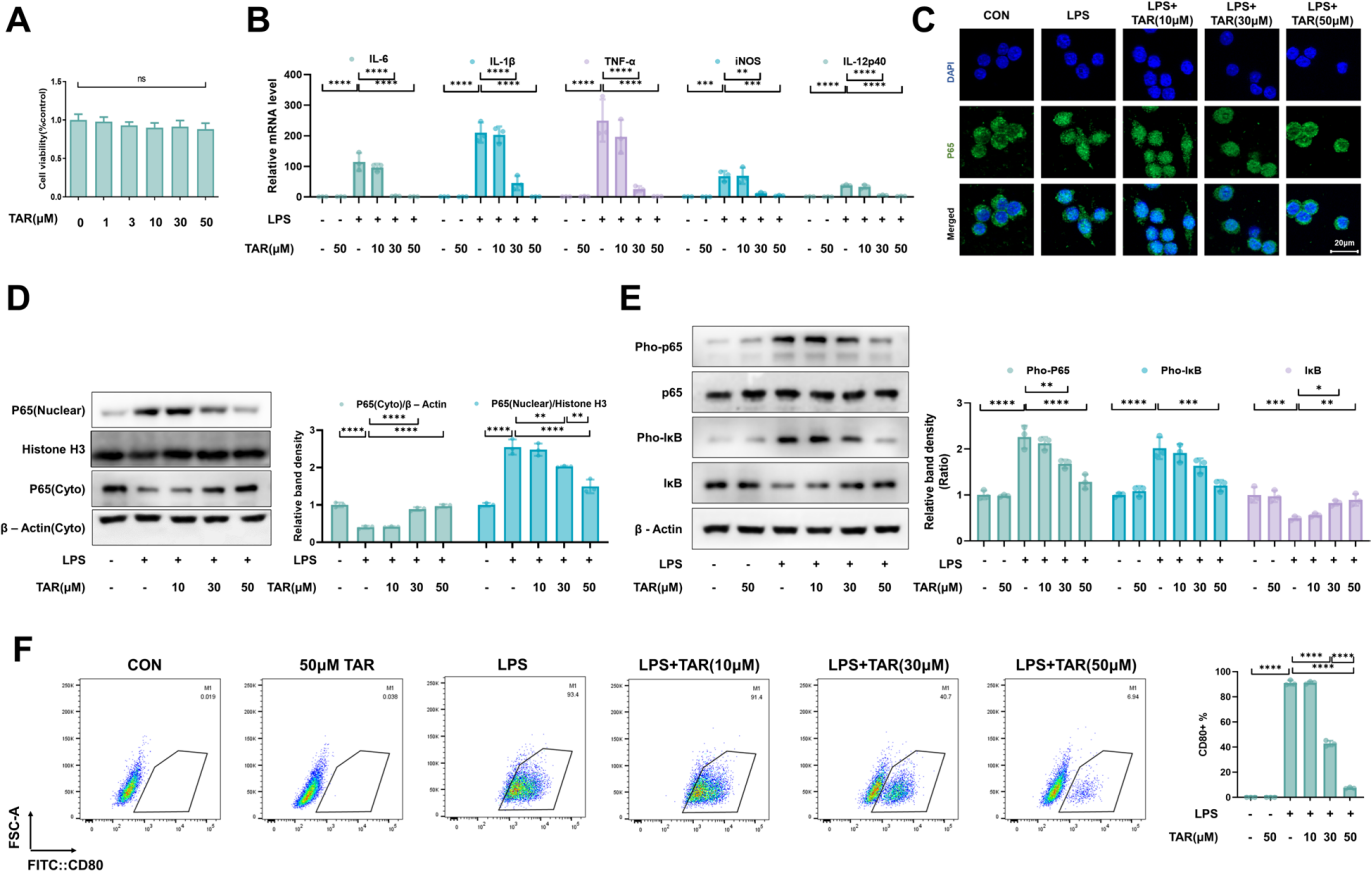
Taraxerone alleviates LPS-induced inflammatory responses in primary macrophages. **A** Primary macrophages were exposed to different concentrations of taraxerone (0, 1, 3, 10, 30, and 50 μM) for 12 h, and cell viability was detected with CCK8 kit. Primary macrophages were exposed to different concentrations of taraxerone (10, 30, 50 μM) and LPS (1 μg/mL) for 12 h. **B** mRNA levels of inflammatory cytokines (IL-1β, IL-6, TNF-α, iNOS, and IL-12) in primary macrophages were detected 12 h later. **C** P65 nuclear translocation was discovered using immunofluorescence staining. The bar represented 20 μm. **D** Western blotting was used to determine the location of P65 in the nuclei and cytoplasm. **E** Western blotting detected Pho-P65, P65, Pho-IκB, and IκB protein levels in primary macrophages. **F** The M1 macrophage proportion was detected using flow cytometry. Data are presented as mean ± SD (n = 3 in each group). *P < 0.05, **P < 0.01, ***P < 0.001, ****P < 0.0001

We looked further into the possibility that taraxerone could reduce the inflammatioin that induced by LPS in primary macrophages based on the outcomes of the above in vivo experiments. In this study, we used a Cell Counting Kit-8 (CCK8) to determine the effects of different doses of taraxerone on cell viability, and the results showed that taraxerone (10, 30, and 50 μM) had no toxic effect on primary macrophages (Fig. 3A). Therefore, primary macrophages were treated with 10, 30, and 50 μM taraxerone and LPS (1 μg/mL) for 12 h. The outcomes showed that LPS markedly increased the mRNA expression of M1-related genes (IL-6, IL-1β, TNF-α, iNOS, and IL-12) in primary macrophages, while 30 and 50 μM of taraxerone markedly inhibited the increase (Fig. 3B). To regulate these M1-related genes, nuclear translocation of NF-κB P65 is necessary. We observed the effect of LPS and taraxerone treatments on the nuclear translocation of P65 in primary macrophages using an immunofluorescence assay. We found that a dose of 50 μM of taraxerone suppressed P65 nuclear translocation induced by LPS (Fig. 3C). Consistent with these results, P65 protein content was markedly increased in the nucleus of primary macrophages treated with LPS alone, which was blocked by treatment with 30 and 50 μM taraxerone (Fig. 3D). In addition, western blotting results showed that LPS treatment increased and decreased the protein expression of Pho-P65 and Pho-IκB in primary macrophages, respectively. In contrast, 50 μM taraxerone could inhibit the effects (Fig. 3E). To observe the effect of taraxerone on the polarization of macrophages, we detected the proportion of CD80 + cells using flow cytometry, and the results showed that taraxerone could inhibit the polarization of M1 macrophages (Fig. 3F). Taken together, taraxerone blocked the NF-κB pathway and inhibit the inflammatory response of primary macrophages induced by LPS.

### Taraxerone alleviates LPS-induced oxidative stress in primary macrophages

**Fig. 4:**
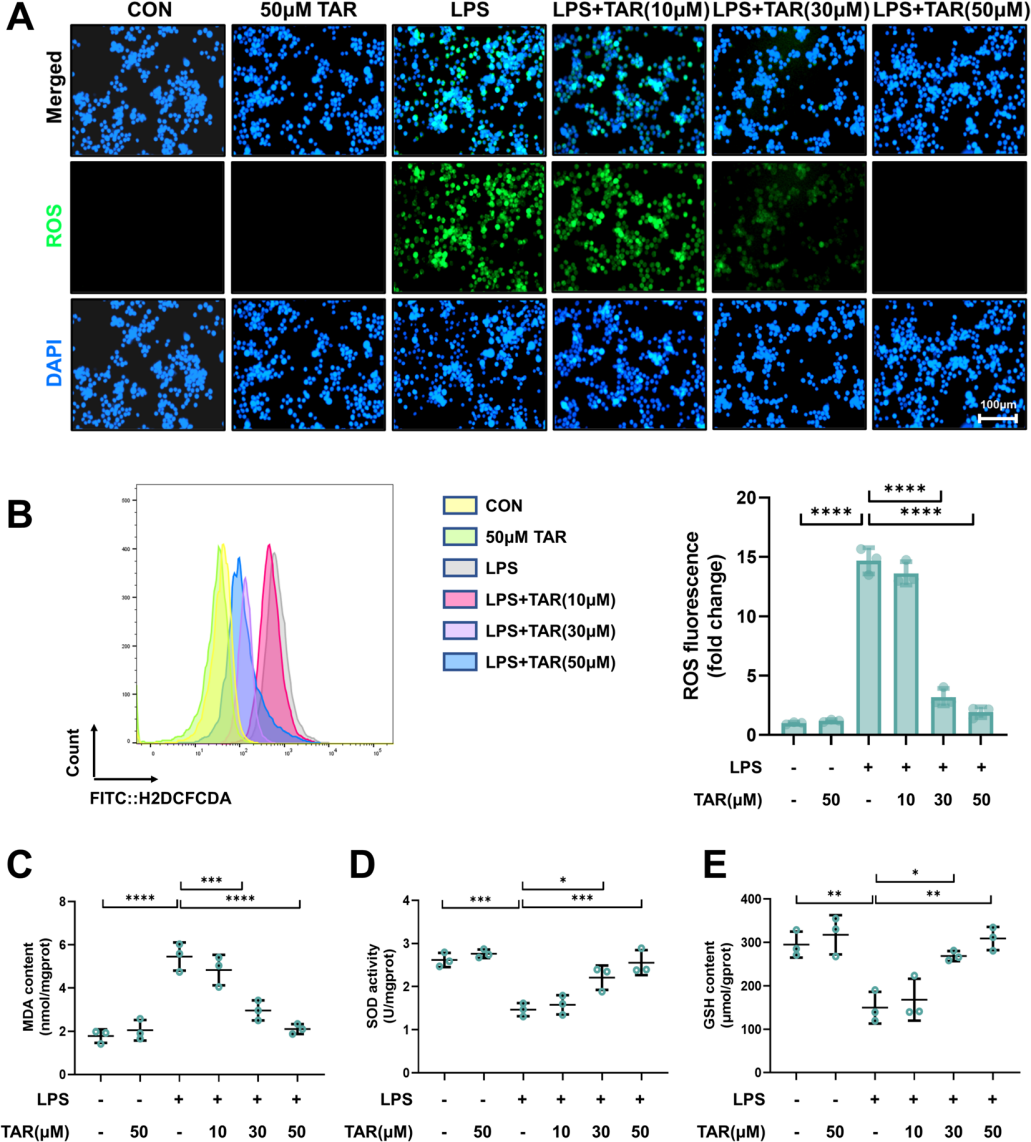
Anti-oxidative stress effect of taraxerone in primary macrophages. Primary macrophages were treated with different concentrations of taraxerone (10, 30, and 50 μM) and LPS (1 μg/mL) for 12 h. **A** Primary macrophages were incubated with H2DCFDA for 30 min to determine the ROS level. The bar represented 100 μm. **B** The ROS level in primary macrophages was detected using flow cytometry, and the data were analyzed using FlowJo. **C**–**E** MDA content, SOD activity, and GSH content were determined. Data are presented as mean ± SD (n = 3 in each group). *P < 0.05, **P < 0.01, ***P < 0.001, ****P < 0.0001

We investigated whether taraxerone protects against LPS-induced oxidative stress in primary macrophages. Our results showed that taraxerone effectively reduced LPS-induced ROS production in primary macrophages (Fig. 4A, B). In accord with the outcomes in vivo, the LPS-induced increase in MDA content in primary macrophages was restrained by taraxerone (Fig. 4C). Additionally, taraxerone treatment markedly prevented the LPS-induced depletion of GSH and inhibition of SOD activity (Fig. 4D, E). These results suggest that taraxerone reduced LPS-induced oxidative damage in primary macrophages.

### Taraxerone inhibits NLRP3 inflammasome activation in vivo and in vitro

**Fig. 5:**
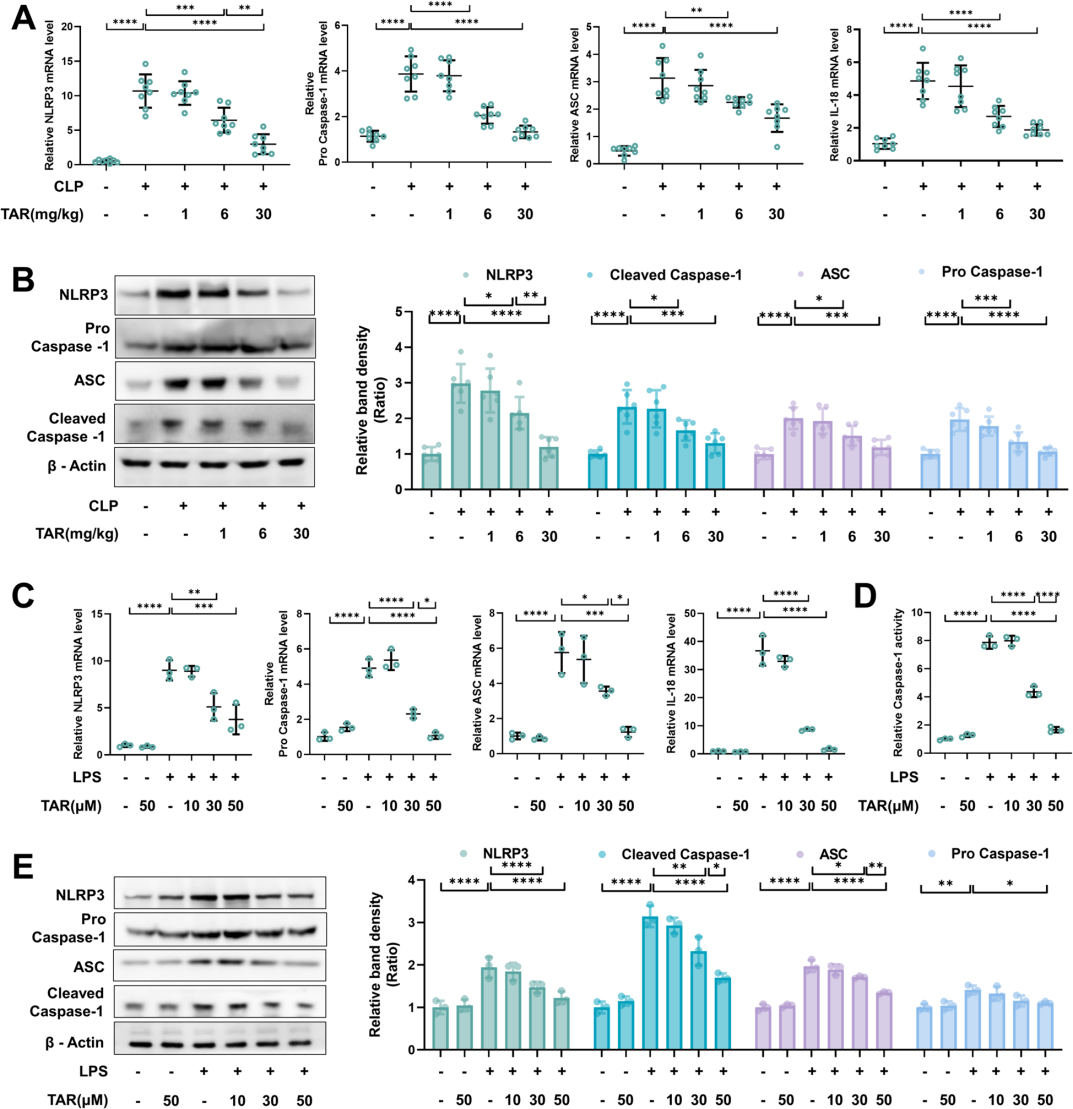
Taraxerone inhibits NLRP3 inflammasome activation in vivo and in vitro. **A**, **B** Mice were pretreated with taraxerone (1, 6, and 30 mg/kg) or saline for 2 h before CLP. The mRNA levels of NLRP3, Pro Caspase-1, ASC, and IL-18 were measured 12 h later. The protein levels of NLRP3, Cleaved Caspase-1, ASC, and Pro Caspase-1 were detected by western blotting. **C** Primary macrophages were treated with different concentrations of tar (10, 30, and 50 μM) and LPS (1 μg/mL). The mRNA levels of NLRP3, Pro Caspase-1, ASC, and IL-18 were measured 12 h later. **D** The activity of Caspase-1 was detected using the assay kit. **E** The protein levels of NLRP3, Cleaved Caspase-1, ASC, and Pro Caspase-1 were detected by western blotting. Data are presented as mean ± SD (n ≥ 6 in vivo, n = 3 in vitro). *P < 0.05, **P < 0.01, ***P < 0.001, ****P < 0.0001

The NLRP3 inflammasome plays an important role in inflammatory response in ALI. Therefore, this study investigated the effects of taraxerone on the NLRP3 inflammasome. Lung tissues were ground in sepsis-induced ALI mice, and protein and RNA were extracted and analyzed. The results showed that sepsis significantly promoted NLRP3, Pro Caspase-1, ASC and IL-18 mRNA levels, increased NLRP3, Pro Caspase-1, Cleaved Caspase-1 and ASC protein expression, whereas pretreatment with 6 and 30 mg/kg taraxerone attenuated these effects (Fig. 5A, B). Similarly, in primary macrophages, 30 and 50 μM taraxerone inhibited LPS-induced NLRP3, Pro Caspase-1, ASC and IL-18 mRNA and protein expression increases, and inhibited the Cleaved Caspase-1 protein expression (Fig. 5C, E). Meanwhile, we detected the Caspase-1 activity of treated cells, the results showed that taraxerone could markedly reduce the activity of Caspase-1 (Fig. 5D). In summary, these results suggest that taraxerone inhibited the activation of the NLRP3 inflammasome in vitro and in vivo.

### Taraxerone regulates SIRT1/NF-κB-NLRP3 inflammasome signaling pathway in primary macrophages

**Fig. 6:**
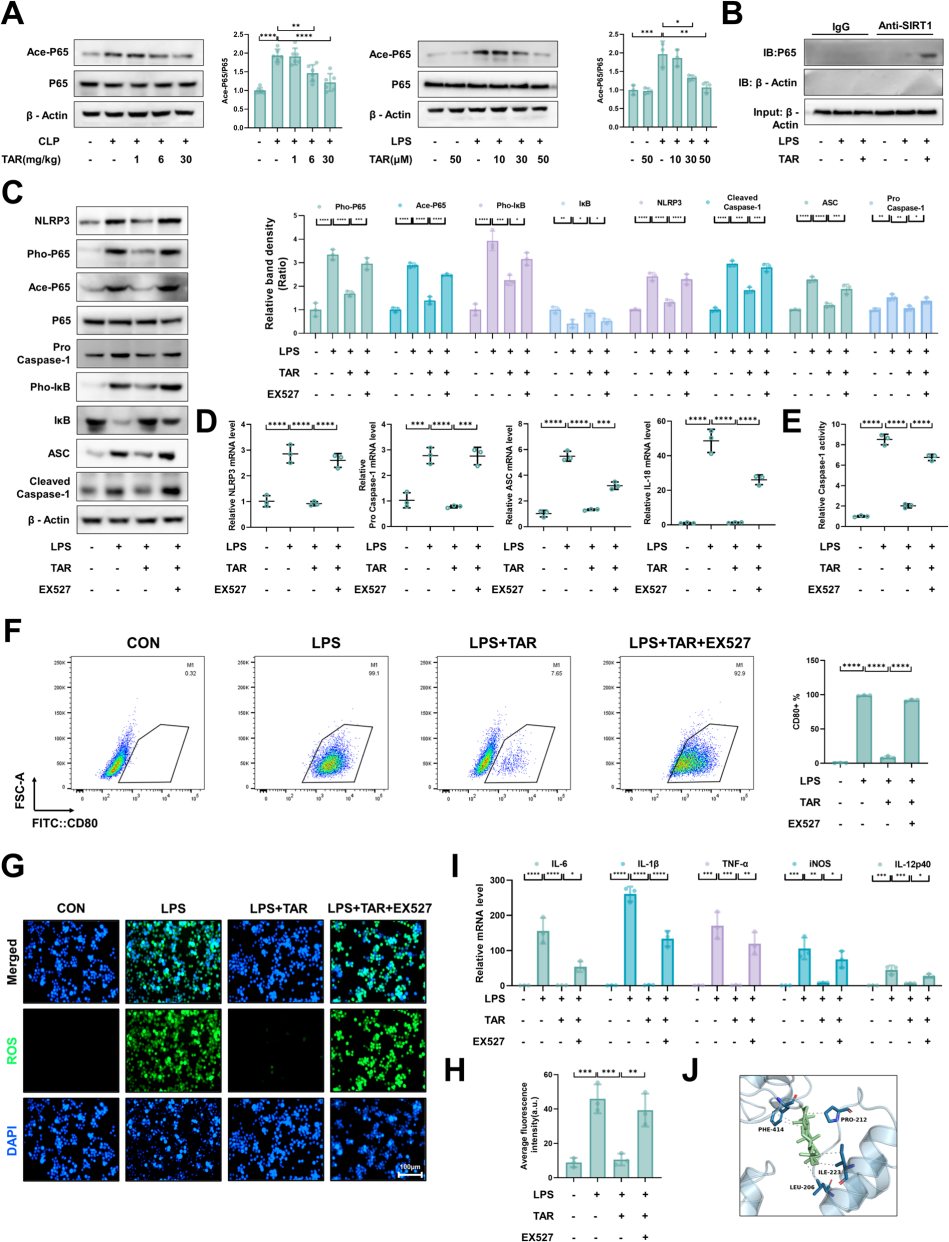
The anti-inflammatory and anti-oxidative stress effects of taraxerone in primary macrophages are exerted by regulating SIRT1. **A** Mice were pretreated with taraxerone (1, 6, and 30 mg/kg) or saline for 2 h before cecal ligation and puncture. Primary macrophages were treated with taraxerone (10, 30, and 50 μM) and LPS (1 μg/mL). The protein levels of Ace-P65 were detected using western blotting. **B** The interaction between SIRT1 and P65 was analyzed using CO-IP. **C** Western blotting was preformed to determine the effects of taraxerone (50 μM) and EX527 (5 μM/mL) on NLRP3, Cleaved Caspase-1, Pro Caspase-1, ASC, Pho-P65, Ace-P65, P65, Pho-IκB, and IκB protein levels. Primary macrophages were pretreated with EX527 (5 μM/mL) for 2 h, and then treated with LPS (1 μg/mL) and taraxerone (50 μM) for 12 h. **D** Detection of NLRP3, Pro Caspase-1, ASC, and IL-18 mRNA levels. **E** The activity of Caspase-1 was measured. **F** The proportion of CD80 + macrophages was detected using flow cytometry. **G**, **H** H2DCFDA probe was used to detect ROS levels. The bar represented 100 μm. **I** Detection of IL-6, IL-1β, TNF-α, iNOS, and IL-12 mRNA levels. **J** Detailed view of protein-tar interaction. The SIRT1 residues involved in the interaction are represented by a stick model, where hydrogen bonds are represented by dashed lines. Taraxerone is also represented by a stick model. Data are presented as mean ± SD (n = 6 in vivo, n = 3 in vitro). *P < 0.05, **P < 0.01, ***P < 0.001, ****P < 0.0001

Acetylation and phosphorylation of NF-κB together maintain nuclear translocation and transcription of NF-κB [37]. Taraxerone effectively reduced the protein expression of Ace-P65 in vitro and in vivo (Fig. 6A). Since the sirtuin family is known for deacetylation, and SIRT1 can deacetylate P65 to play an vital role in many anti-inflammatory and anti-oxidative stress pathways, we investigated whether taraxerone could activate SIRT1 to regulate the NF-κb-NLRP3 inflammasome pathway. We examined the interaction between SIRT1 and P65 by co-immunoprecipitation, and the results showed that SIRT1 directly combined with P65 to play a role (Fig. 6B). Primary macrophages were treated with EX527 (5 μM/mL) 2 h in advance, and then treated with taraxerone (50 μM) and LPS (1 μg/mL) for 12 h to detect relevant indicators. Compared with the LPS and taraxerone treatment groups, pretreatment with EX527 blocked the effects of taraxerone on protein expression levels (Fig. 6C). Pretreatment with EX527 also markedly suppressed the inhibitory effect of taraxerone on the LPS-induced increase in the mRNA expression of NLRP3, Pro Caspase-1, ASC, and IL-18, and the activity of Caspase-1 (Fig. 6D, E). Besides, we observed the effect of EX527 on macrophages polarization in vitro. The results showed that EX527 restrained the inhibitory effect of taraxerone on M1 macrophage polarization (Fig. 6F). In addition, ROS level in primary macrophages was measured, and the results showed that 50 μM taraxerone had an inhibitory effect on excessive ROS, but this effect was significantly limited by EX527 pretreatment (Fig. 6G, H). Consistent with this, tarxerone inhibited the LPS-induced increase of M1-related gene mRNA expression, and EX527 suppressed this effect (Fig. 6I). Because taraxerone prevented M1 macrophage polarization by activating SIRT1, we investigated whether taraxerone could directly bind to SIRT1 using molecular docking. The results showed that taraxerone may have direct effect on SIRT1 (Fig. 6I). These results indicate that the anti-inflammatory and anti-oxidative stress effects of taraxerone on primary macrophages were regulated by SIRT1.

### SIRT1 mediated the protective effects of taraxerone on sepsis-induced ALI mice

**Fig. 7:**
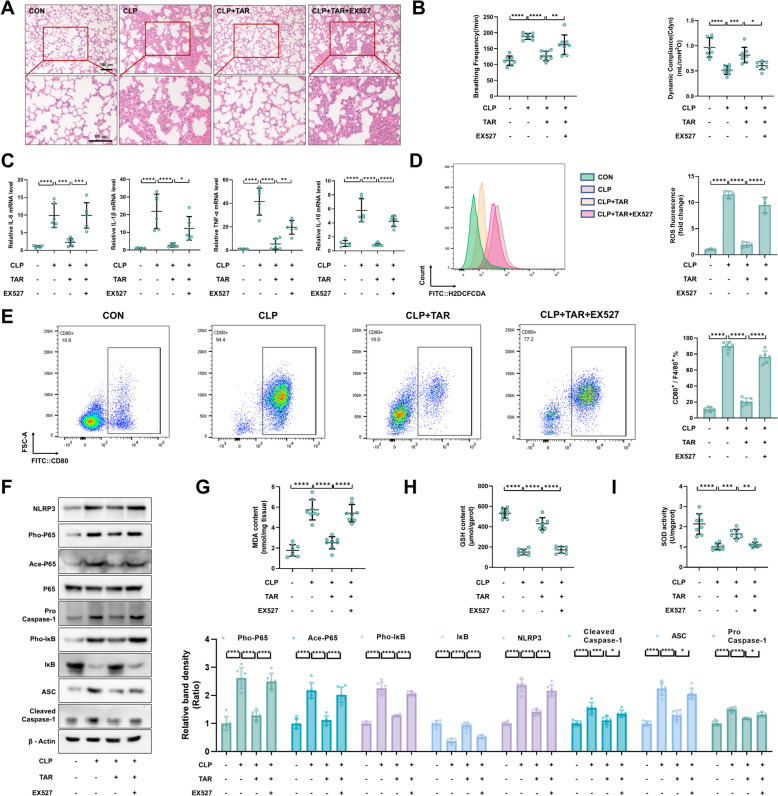
Taraxerone plays a protective role in sepsis-induced ALI mice through the SIRT1 pathway. The EX527 (10 mg/kg) was administered intraperitoneally 2 h before the taraxerone (0, 30 mg/kg) treatment, and the lung tissues and BALFs were collected after the CLP model was constructed 12 h later. **A** With H&E staining, we examined the lung tissue for any pathological changes. The bar represented 100 μm. **B** The breathing frequency and dynamic compliance of mice were detected by BUXCO system. **C** Detection of IL-1β, IL-6, TNF-α, and IL-18 mRNA levels. **D** ROS level in BALFs were determined using flow cytometry and analyzed with FlowJo. **E** Changes in the proportion of M1 macrophages in BALFs were detected using flow cytometry, and the data were analyzed using FlowJo. **F** Effects of taraxerone (30 mg/kg) and EX527 (10 mg/kg) on NLRP3, Cleaved Caspase-1, Pro Caspase-1, ASC, Pho-P65, Ace-P65, P65, Pho-IκB, and IκB protein levels. **G**–**I** MDA content, GSH content, and SOD activity were determined to reflect the oxidative stress levels of lung tissues. Data are presented as mean ± SD (n ≥ 6 in each group). *P < 0.05, **P < 0.01, ***P < 0.001, ****P < 0.0001

To verify whether the protective effects of taraxerone were mediated by SIRT1 activation, EX527, a specific inhibitor of SIRT1, was injected 2 h before taraxerone treatment. Taraxerone did not reduce tissue damage or edema in mice with ALI in the presence of EX527 (10 mg/kg) (Fig. 7A). Besides, we determined the protective effect of taraxerone on sepsis-induced mice were restrained by EX527 through the test of pulmonary function (Fig. 7B). The mRNA levels of cytokines (IL-6, IL-1β, TNF-α) and IL-18 also presented the same results (Fig. 7C). The inhibitory effect of taraxerone on ROS production was limited by EX527 treatment (Fig. 7D). In addition, we examined the changes in the proportion of M1 macrophages. Flow cytometry showed that taraxerone inhibited the increase in M1 macrophages in sepsis-induced ALI mice, whereas EX527 blocked this effect (Fig. 7E). Furthermore, the presence of EX527 significantly limited the inhibitory effect of taraxerone on the NF-κB-NLRP3 inflammasome pathway (Fig. 7F). Consistent with the results of the in vitro experiments, taraxerone treatment markedly decreased the MDA content and increased the levels of GSH and SOD activity, but this effect was greatly inhibited in the presence of EX527 (Fig. 7G–I). In summary, taraxerone inhibited inflammatory damage and oxidative stress in sepsis-induced ALI mice by activating SIRT1.

## Discussion

Our study revealed, for the first time, that taraxerone may directly bind to and activate SIRT1 in macrophages. This activation reduced the phosphorylation and acetylation of p65, thereby inhibiting the activation of the NF-κB signaling pathway. Simultaneously, the NLRP3 inflammasome signaling pathway was also blocked. Consequently, in vitro, taraxerone alleviated LPS-induced ROS-mediated M1 polarization of macrophages, while in vivo, it reduced sepsis-induced inflammatory response and pulmonary oxidative stress. This suggested that taraxerone may serve as a potential drug targeting SIRT1 for the treatment of sepsis-induced acute lung injury.

When ALI develops, inflammatory cells trigger inflammatory response and ROS production, and oxidative stress amplifies the expression of pro-inflammatory genes, creating a vicious cycle that plays a vital role in the development of ALI [30, 38]. In this study, we first demonstrated that taraxerone activates SIRT1 both in vitro and in vivo, alleviating oxidative stress and the inflammatory response in the lungs of mice with sepsis-induced ALI.

Inflammatory cells in the lung tissue are continually stimulated during sepsis-induced ALI, with macrophage polarization playing a crucial role [39]. The main pathways that stimulate macrophage polarization toward M1 phenotype are the JAK/STAT pathway, PI3K/AKT2 pathway, and MyD88/TRIF pathway, all of which regulate M1 genes by activating NF-κB, promoting degradation of inhibitor kappa-B(IκB) and translocation of P65 into the nucleus [14, 40]. This process promotes the polarization of macrophages towards the M1 phenotype and causes them to release massive inflammatory cytokines like IL-6, IL-1β, IL-12, and TNF-α [41–43]. Sequestering the coactivators of NF-κB suppresses the activation of M1 targets and limits M1 polarization [44]. When macrophages polarize to the M1 phenotype, they recruit neutrophils, monocytes, and other inflammatory cells to enhance the inflammatory response. Prolonged and massive activation of M1 macrophages causes tissue and organ damage, thus aggravating lung injury [10]. In our study, taraxerone inhibited sepsis-induced increases in inflammatory cytokines like TNF-α, IL-1β, and IL-6. Taraxerone also reduced pulmonary edema, lowered the W/D ratio, suppressed the MPO activity, and decreased the counts of inflammatory cells and the proportion of macrophages with the M1 phenotype. Moreover, the results showed that taraxerone had the same effect of suppressing the inflammatory response in LPS-treated primary macrophages.

Furthermore, macrophage activation in the lungs promotes inflammatory cytokines and cells release and increases intracellular ROS production, which is highly correlated with the severity of ALI [30, 45]. Numerous studies have shown that excessive ROS production can increase NF-κB activity, causing damage to the structure of lung tissue [46, 47]. By enhancing the activation of the NF-κB signaling pathway, ROS are also involved in promoting the inflammatory response of alveolar macrophages [48–50]. The activation of NF-κB regulates the expression of M1-related genes (such as iNOS), and the upregulation of iNOS leads to the aggravation of the oxidative stress injury. Moreover, NF-κB receives signals generated by excessive ROS, thereby increasing the protein levels of NLRP3, completing the activation and assembly of NLRP3 inflammasome [51, 52]. Additionally, NLRP3 inflammasome assembly results in self-cleavage and activation of Pro Caspase-1, maturing IL-1β and IL-18, further exacerbating inflammation [53]. Hosseinian and Huang have reported that the NLRP3 inflammasome plays a significant role in the ALI progression [54, 55]. Our results consistently showed that the levels of the NLRP3 inflammasome in vivo and i*n vitro* were elevated upon induction with CLP or LPS and that pretreatment with taraxerone markedly inhibiedt inflammatory amplification and oxidative stress. Excessive production of MDA and ROS in the lungs can be inhibited by the antioxidant enzymes SOD and GSH, thereby attenuating oxidative damage and protecting cells [56, 57]. Our findings demonstrated that taraxerone markedly inhibited the increase in ROS and MDA levels in both sepsis-induced ALI mice and LPS-induced primary macrophages, whereas SOD activity and GSH levels were prominently increased by taraxerone treatment. According to Zhang et al., M1 macrophage polarization can be promoted by excessive ROS production in an inflammatory environment and is limited by ROS scavengers [58]. This is consistent with the results of our study, in which taraxerone treatment inhibited excessive ROS production and oxidative stress caused by sepsis.

Histone deacetylases (HDACs) have a wide range of deacetylating effects on various proteins. The sirtuin family is known as class III histone deacetylase [59]. SIRT1 is a crucial factor in anti-inflammatory and anti-oxidative stress [60, 61]. After SIRT1 activation, RelA/P65 deacetylation occurs at Lys-310, which reduces the level of NF-κB P65 acetylation, inhibits the transcriptional activity of the NF-κB complex, and decreases the levels of inflammatory factors [23]. By preventing NF-κB from fully activating, SIRT1 further reduces the M1 phenotypic polarization of macrophages and increases the M2 phenotype polarization in different disease models [62, 63]. Our previous studies have shown that taraxerone can regulate the process of myoblast differentiation by regulating SIRT1 [64]. Several studies have found that SIRT1 alleviates ALI by improving the epithelial barrier function and reducing the permeability of endothelial tight junctions [65, 66]. The main effect of SIRT1 on NF-κB P65 is deacetylation, which suppresses its transcriptional activity. SIRT1 decreases the activation and assembly of NLRP3 inflammasome and reduces the levels of inflammatory cytokines to attenuate the inflammatory response through the inhibition of NF-κB P65 activity [21, 67, 68]. In addition, we verified through co-immunoprecipitation that taraxerone enhances SIRT1 deacetylation by directly binding to P65. Our results showed that SIRT1 inhibits the phosphorylation of P65, limiting the nuclear translocation of P65. The dephosphorylation effect of SIRT1 may be achieved by activation of downstream kinases or by SIRT1 sulfation, which can restrain the activation of NF-κB P65 and reduce its nuclear translocation [69–71]. Studies have established the pivotal role of SIRT1 in acute lung injury [65]. However, the majority of compounds exert their effects through the SIRT1 pathway without direct binding and activation. In our experiments, taraxerone appears to function as a direct activator of SIRT1, presenting a unique mechanism compared to many other compounds in the studies. However, the specific molecular mechanisms underlying the inhibitory effect of SIRT1 on P65 phosphorylation require further investigation. Moreover, Chan et al. found that inhibiting SIRT1 aggravated oxidative stress in coronary monocytes [72]. Yin et al. revealed that the deficiency of SIRT1 in the liver leads to a remarkable increase in intrahepatic ROS levels, resulting in severe oxidative stress [73]. The anti-oxidative stress effect of SIRT1 is exerted through various pathways; for example, SIRT1 reduces the excessive production of ROS by upregulating SOD activity or inhibiting NF-κB activity [74]. In our study, the SIRT1 inhibitor EX527 and taraxerone were used successively to treat mice and primary macrophages, and the results showed that EX527 blocked taraxerone-inhibited sepsis-induced or LPS-induced elevated ROS levels. These results showed that the anti-inflammatory and anti-oxidative stress effects of taraxerone were limited by EX527 treatment and that the effects of taraxerone in vivo and in vitro were mediated via SIRT1. Based on the above results, we found that taraxerone could activate SIRT1; therefore, we further conducted molecular docking to detect the interaction between SIRT1 and taraxerone. These two may connect with hydrogen bonds at PHE-414, PRO-212, ILE-223, and LEU-206 of SIRT1.Therefore, the results showed that taraxerone may act directly on SIRT1.

## Conclusion

**Fig. 8:**
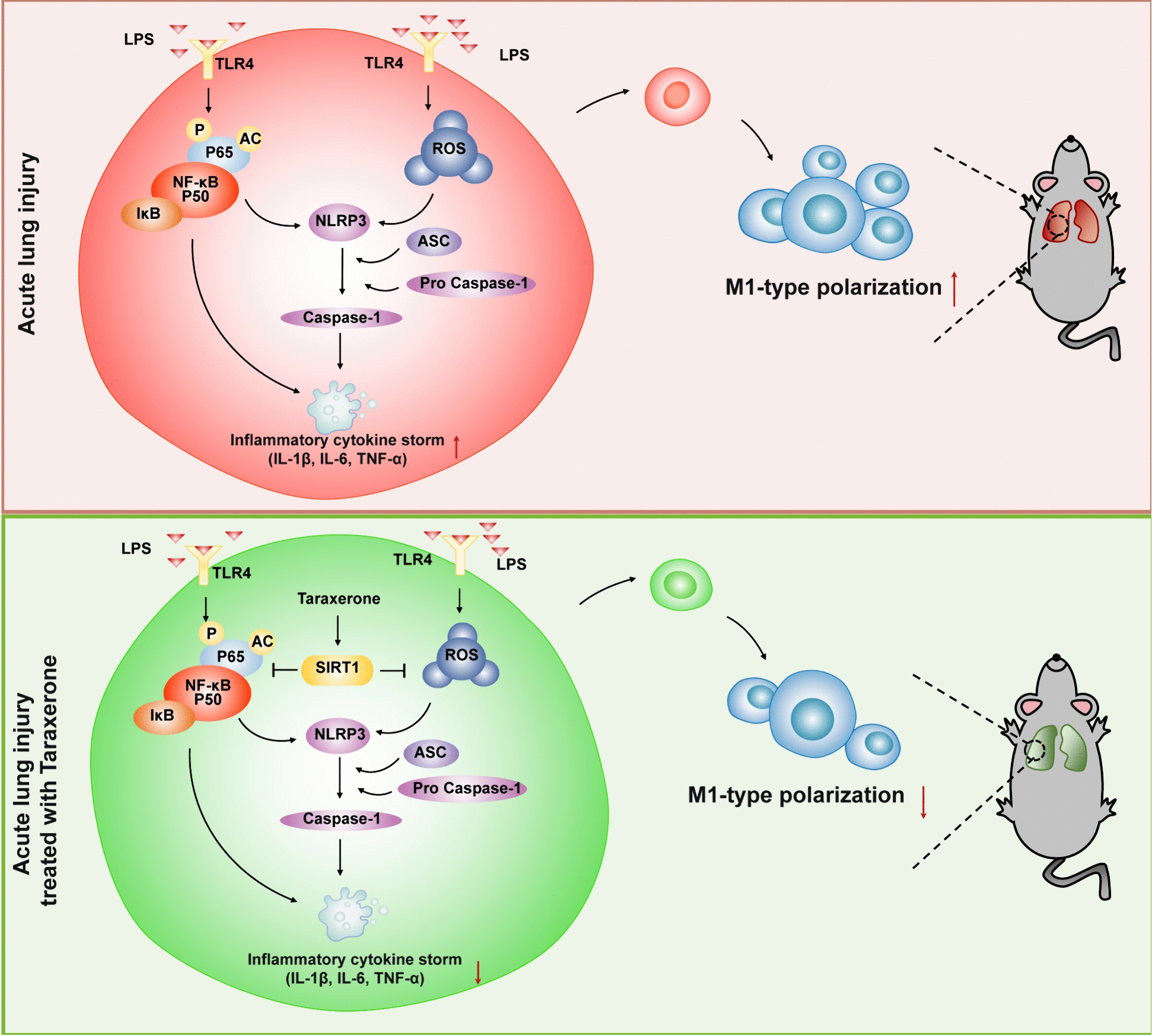
Taraxerone plays a protective role in acute lung injury by activating SIRT1. Taraxerone activates SIRT1 to block the NF-κB-NLRP3 inflammasome axis and to suppress the excessive ROS, inhibiting the M1 polarization of macrophages, and exerting a protective effect on acute lung injury mice.

Our results demonstrate for the first time that taraxerone exhibits a significant inhibitory effect on inflammation and oxidative stress in sepsis-induced ALI mice that was dependent on the activation of SIRT1 via inhibiting the NF-κB-NLRP3 inflammasome pathway and suppressing the excessive ROS, as shown in Fig. 8. This special effect of taraxerone warrant further investigation for the treatment of inflammation and oxidative stress-related diseases.

## Supplementary Information

Additional file 1: Fig. S1. A Detection of IL-1β, IL-6, and TNF-α mRNA levels. Data are presented as mean ± SD (n = 6 in each group). ****P < 0.0001. Fig. S2. Mice were pretreated with taraxerone (30 mg/kg), dexamethasone (5 mg/kg) or saline for 2 h before cecal ligation and puncture, and the BALF and lung tissues were collected to analyzed 12 h after the model was established. A Pathological changes in the lungs observed through H&E staining. The bar represented 100 μm. B Pulmonary edema was detected by the measurement of W/D ratio. C, D BALFs were gathered for the detection of total protein content and counts of neutrophils and macrophages in lungs. E Determination of TNF-α, IL-1β, IL-6, and IFN-γ using ELISA. F Measurement of MPO activity in lung. Fig. S3. Mice were pretreated with taraxerone (30 mg/kg) or saline for 2 h before cecal ligation and puncture, and the serum and lung tissues were collected to analyzed 12 h after the surgery. A H&E staining in the spleens, livers, and kidneys of mice. The bar represented 100 μm. B Determination of TNF-α, IL-1β, and IL-6 using ELISA.

## Data Availability

The data used to support the findings of the study are available from the corresponding author on reasonable request.

## Abbreviations

ALI: Acute lung injury
AMs: Alveolar macrophages
ARDS: Acute respiratory distress syndrome
ASC: Apoptosis-associated speck-like protein containing a CARD
BALF: Bronchoalveolar lavage fluid
CLP: Cecal ligation and puncture
Co-IP: Co-Immunoprecipitation
DHE: Dihydroethidium
GSH: Glutathione
HDACs: Histone deacetylases
H&E: Hematoxylin&eosin
IL-1β: Interleukin-1 beta
IL-6: Interleukin-6
IL-12: Interleukin-12
iNOS: Inducible nitric-oxide synthase
LPS: Lipopolysaccharide
MDA: Malondialdehyde
MPO: Myeloperoxidase
MyD88: Myeloid differentiation primary response gene (88)
NF-κB: Nuclear factor kappa-B
NLRP3: NACHT, LRR and PYD domains-containing protein 3
ROS: Reactive oxygen species
SIRT1: Sirtuin 1
SOD: Superoxide dismutase
Tar: Taraxerone
TLR4: Toll-like receptor 4
TNF-α: Tumor necrosis factor-alpha
W/D: Wet/dry
