# Supplementary material for: Taraxerone inhibits M1 polarization and alleviates sepsis-induced acute lung injury by activating SIRT1
Source: Chin Med. 2024 Nov 14;19:159. doi: 10.1186/s13020-024-01002-z (PMC11566926; doi:10.1186/s13020-024-01002-z)
Supplement: Supplementary file 1 — Additional file 1: Fig. S1. A Detection of IL-1β, IL-6, and TNF-α mRNA levels. Data are presented as mean ± SD (n = 6 in each group). ****P < 0.0001. Fig. S2. Mice were pretreated with taraxerone (30 mg/kg), dexamethasone (5 mg/kg) or saline for 2 h before cecal ligation and puncture, and the BALF and lung tissues were collected to analyzed 12 h after the model was established. A Pathological changes in the lungs observed through H&E staining. The bar represented 100 μm. B Pulmonary edema was detected by the measurement of W/D ratio. C, D BALFs were gathered for the detection of total protein content and counts of neutrophils and macrophages in lungs. E Determination of TNF-α, IL-1β, IL-6, and IFN-γ using ELISA. F Measurement of MPO activity in lung. Fig. S3. Mice were pretreated with taraxerone (30 mg/kg) or saline for 2 h before cecal ligation and puncture, and the serum and lung tissues were collected to analyzed 12 h after the surgery. A H&E staining in the spleens, livers, and kidneys of mice. The bar represented 100 μm. B Determination of TNF-α, IL-1β, and IL-6 using ELISA. [file 13020_2024_1002_MOESM1_ESM.docx]

**
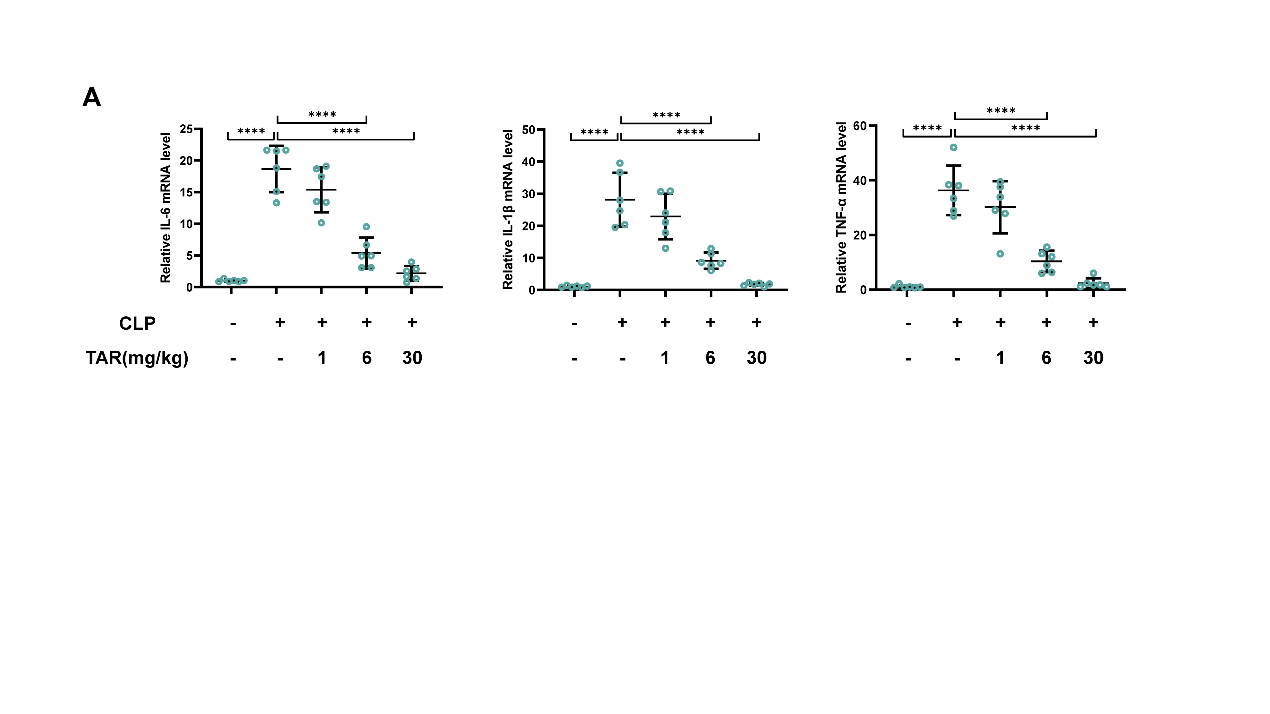
**

**Fig. S1.** (A) Detection of IL-1β, IL-6, and TNF-α mRNA levels. Data are presented as mean ± SD (n = 6 in each group). *****P* < 0.0001.

**
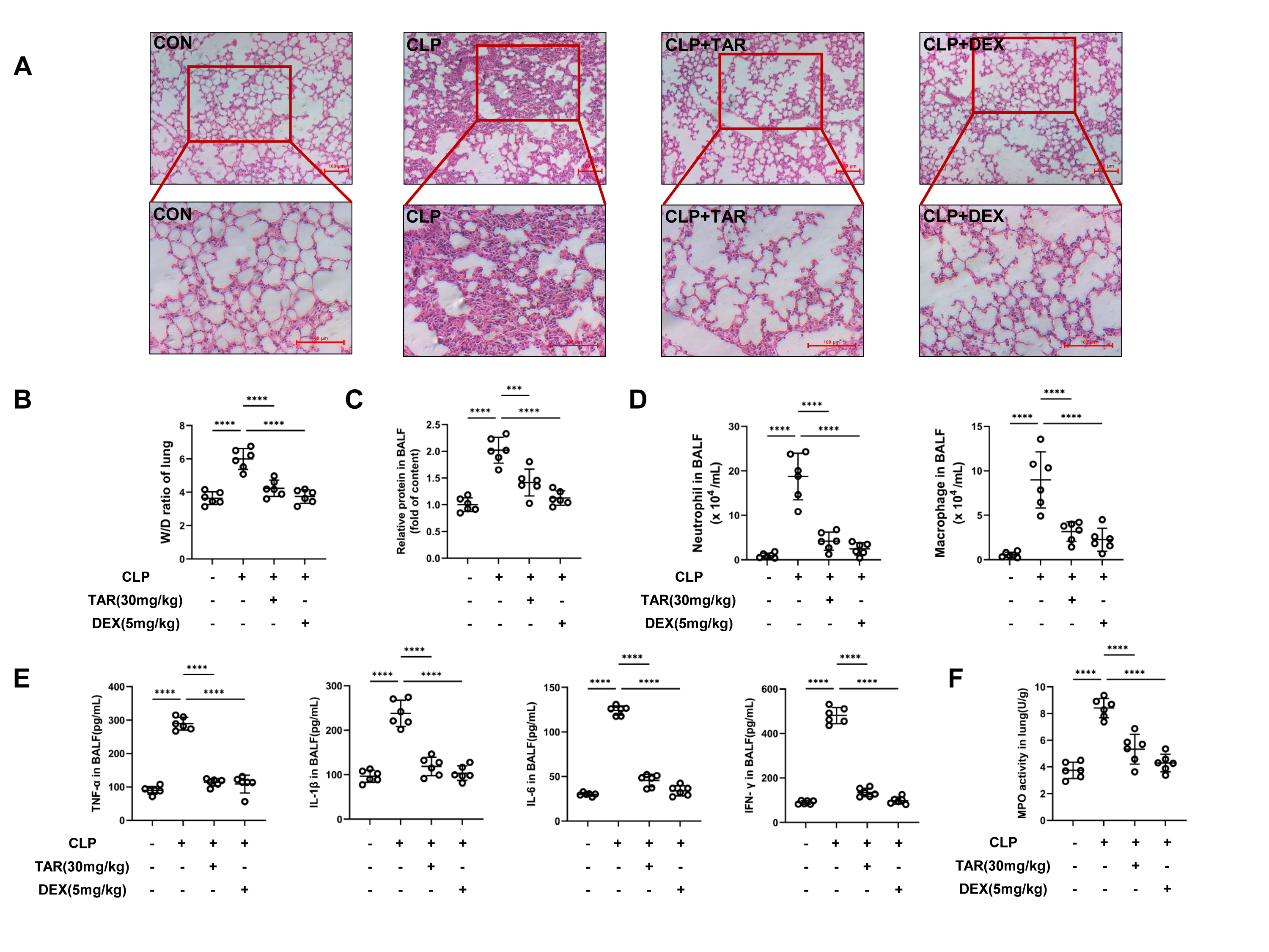
**

**Fig. S2.** Mice were pretreated with taraxerone (30 mg/kg), dexamethasone (5 mg/kg) or saline for 2 h before cecal ligation and puncture, and the BALF and lung tissues were collected to analyzed 12 h after the model was established. (A) Pathological changes in the lungs observed through H&E staining. The bar represented 100 μm. (B) Pulmonary edema was detected by the measurement of W/D ratio. (C - D) BALFs were gathered for the detection of total protein content and counts of neutrophils and macrophages in lungs. (E) Determination of TNF-α, IL-1β, IL-6, and IFN-γ using ELISA. (F) Measurement of MPO activity in lung.


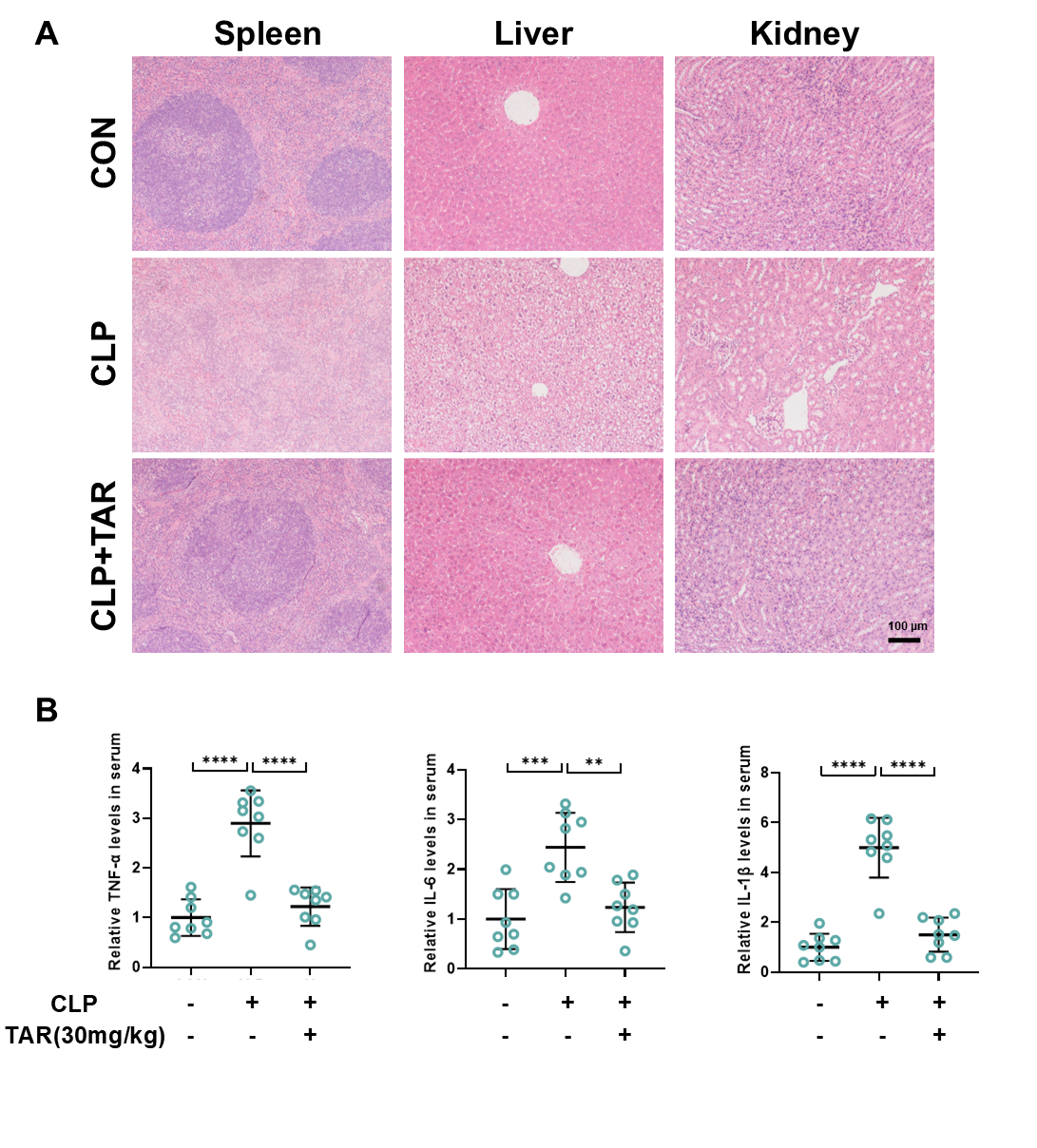


**Fig. S3.** Mice were pretreated with taraxerone (30 mg/kg) or saline for 2 h before cecal ligation and puncture, and the serum and lung tissues were collected to analyzed 12 h after the surgery. (A) H&E staining in the spleens, livers, and kidneys of mice. The bar represented 100 μm. (B) Determination of TNF-α, IL-1β, and IL-6 using ELISA.
